# Relationship between 8-year weight change, body size, and health in a large cohort of adults in Thailand

**DOI:** 10.1016/j.je.2016.09.015

**Published:** 2017-06-17

**Authors:** Vasoontara Yiengprugsawan, Wimalin Rimpeekool, Keren Papier, Cathy Banwell, Sam-ang Seubsman, Adrian C. Sleigh

**Affiliations:** a Centre for Research on Ageing, Health and Wellbeing, Research School of Population Health, Australian National University, Canberra, Australia; b Australian Research Council Centre of Excellence on Population Ageing Research, Canberra, Australia; c School of Human Ecology, Sukhothai Thammathirat Open University, Nonthaburi, Thailand; d National Centre for Epidemiology and Population Health, Research School of Population Health, Australian National University, Canberra, Australia

**Keywords:** Weight change, Body mass index, Health, Quality of life, Cohort, Thailand

## Abstract

**Background:**

Overweight and obesity have been shown to be risk factors for a range of non-communicable diseases, especially cardio-metabolic conditions, worldwide. But less is known about the effects of weight change on adults' overall health and wellbeing, particularly in transitional low- and middle-income countries. This study aimed to assess the relationship between 8-year weight change and measures of self-assessed health among Thai adults.

**Methods:**

Data were collected from Thai adults aged 25–40 years (n = 27,003) enrolled in the Thai cohort Study and surveyed in 2005, 2009, and 2013. We used self-reported weight and height measurements at baseline and 2013, as well as three standard health questions regarding overall health, energy, and emotion asked at the two time points, to investigate the effects of weight change on health.

**Results:**

Between 2005 and 2013, 6.0% of participants lost more than 5% of their baseline weight; 38.5% were stable (<5% loss to 5% gain); 23.0% slightly gained weight (>5%–10%); 22.8% gained moderate weight (>10%–20%); and 9.4% had heavy weight gain (>20%). Moderate (>10%–20%) and heavy weight gain (>20%) were both associated with an increased risk of reporting ‘poor or very poor‘ overall health in 2013 among participants who had a normal body mass index (BMI) (adjusted odds ratio [AOR] 1.39; 95% confidence interval [CI], 1.13–1.71 and AOR 1.44; 95% CI, 1.09–1.90, respectively), were overweight (AOR 1.53; 955 CI, 1.01–2.29 and AOR 1.82; 95% CI, 1.04–3.19, respectively) or had obesity (AOR 2.47; 95% CI, 1.74–3.51 and AOR 3.20; 95% CI, 2.00–5.16, respectively) in 2005. Weight gain of over 20% also had a negative impact on energy level among cohort members with a normal BMI in 2005 (AOR 1.36; 95% CI, 1.11–1.65) and among participants with obesity in 2005 (AOR 1.93; 95% CI, 1.38–2.71). For those who were underweight, had a normal BMI, or had obesity at baseline, weight loss of more than 5% was associated with reporting emotional problems. Excessive weight gain adversely impacted participants who were underweight or had obesity at baseline.

**Conclusion:**

Our study found that weight change, in particular weight gain, was associated with negative health outcomes, and this effect appeared to increase at higher levels of body size. The present findings may be useful to promote weight maintenance and healthy lifestyles.

## Highlights

• This study investigated 8-year weight change and overall health among Thai adults.
• Weight gain was associated with poor emotional well-being and lower levels of energy.
• Findings highlight the importance of monitoring weight change and health effects.

## Introduction

Increasing weight, overweight, and obesity signal a growing risk of hypertension, high cholesterol, cardiovascular diseases, and other health problems throughout the world. As the global burden of disease shifts to non-communicable diseases (NCDs),^1^ it becomes imperative to understand the relationships between changes in body size and health outcomes. Obesity has been known to have an impact on health and quality of life.^2^, ^3^ However, less is known about the relationship between weight change and indicators of poorer health and quality of life, especially in low- and middle-income countries. Questions still remain about the degree of weight change associated with development of adverse health outcomes and the direction of causality over time.

In Thailand, concern has been growing about increasing obesity and diet-related health risks. Thailand has been among the leaders in Southeast Asia in its rapid urbanization and economic development over the last 3 to 4 decades, which has been accompanied by a move toward more sedentary work, car use, and an increasingly calorie-dense national diet containing more fat, sugar, and salt.^4^, ^5^ National examination health surveys conducted since 1991 (in 1991, 1997, 2004, and 2009) have shown corresponding increases in Thai body weight and in NCDs.^6^, ^7^

This study investigates weight change over time and its relationship with three main health outcomes (self-assessed health, energy, and emotion). The study used data collected from a large sample of adult Open University students undertaking distance learning in Thailand, who reside throughout the country, are well-educated, employed, and of average income. Cohort members' longitudinal weight change over 8 years was used to predict adverse health outcomes during the 8-year follow-up.

## Methods

Our initial research cohort included 87,151 distance-learning adult students enrolled at Sukhothai Thammathirat Open University who were living all over Thailand. They completed the mail-out baseline questionnaire in 2005 investigating transitional patterns of health risks and outcomes. Topics included a range of socio-demographic characteristics, family background, occupation, income, and wellbeing, and health status. The median age at baseline was 29 years, slightly more than half were females, and half resided in urban areas.^8^, ^9^ The cohort was subsequently followed up in 2009 and 2013 (capturing more than 70% at each wave). Analyses presented here were restricted to cohort members aged 25–40 years at the 2005 baseline to limit the otherwise large confounding effect of age on body mass index and health outcomes, resulting in a sample of 27,003 participants.

For our exposure of interest, body mass index (BMI) was derived from weight and height reported at each wave of data collection. We follow the International Obesity Task Force guidelines for BMI cut-offs for Asian populations: BMI 18.5 to <23 as ‘normal’, 23 to <25 as ‘overweight at risk’, and ≥25 as ‘overweight and obese’^10^; these cut-offs have also been used in other studies based on our cohort.^11^, ^12^ Weight change was calculated in 2013, as a percent of 2005 baseline weight, categorized into five percentage groups:

(1) weight loss >5% (loss);
(2) weight loss or gain ≤5% (stable);
(3) weight gain >5% and ≤10% (slight gain);
(4) weight gain >10% and ≤20% (moderate gain);
(5) weight gain >20% (heavy gain)

Three variables from the standardized Medical Outcome Short Form (SF-8) instrument were used to investigate adverse outcomes for the study.^13^ Subjective self-rated health has been applied in international literature to measure overall health and has been shown to be a strong predictor of mortality.^14^, ^15^ In this study, we use three main variables (self-rated health, energy level, and emotion); two of these variables had six possible responses, and one variable had five possible responses. We converted responses for each variable into a binary format as follows: the last two responses were combined into an ‘adverse’ outcome category for all variables, and the first three or four responses were combined into a ‘non-adverse’ health outcome. These adjustments permitted binary analyses. The questions were the same at the 2005 baseline and the 2013 follow-up, as follows:

• *Overall, how would you rate your health during the past 4 weeks*? (excellent, very good, good, or fair = 0; poor or very poor = 1)
• *During the past the past 4 weeks, how much energy did you have*? (very much, quite a lot, a lot, or some = 0; a little or none = 1)
• *During the past 4 weeks, how much have you been bothered by emotional problems (such as feeling anxious, depressed, or irritable)*? (Not at all, slightly, or moderately = 0; quite a lot or extremely = 1)

Initial analyses showed cohort attributes (sex, age, residence, income, and BMI) by 8-year weight change categories. We used 8-year longitudinal weight change to predict health outcomes among cohort members who did not initially report adverse health status at baseline. Analyses were stratified by 2005 BMI categories (underweight, normal, overweight, or obese) in order to investigate associations between weight change and health outcomes according to cohort members' initial body size.

Multivariate logistic analyses of the binary SF-8 health outcomes by 8-year longitudinal weight change were performed to obtain adjusted odds ratios (AORs) and 95% confidence intervals (CIs). Each set of multivariate logistic analyses only included cohort members who had not reported adverse SF-8 health outcomes at baseline (e.g., excluded 1209 for overall health, 2703 for energy level, and 3567 for emotional level). This is also one of the reasons for restricting analyses for cohort members aged 25–40 years, as older cohort members were more likely to be excluded at the baseline. Individuals with missing data for any given analyses were excluded (<5% for each variable), so totals could vary due to available information.

Ethics approval for the overall study was obtained from Sukhothai Thammathirat Open University Research and Development Institute (protocol 0522/10) and the Australian National University Human Research Ethics Committee (protocol 2009/570). Informed written consent was obtained from all participants.

## Results

Among the 27,003 analyzed cohort members, 45% were males (Table 1); at baseline, 38.0% were aged 25–29 years, 30.7% were aged 30–34 years, and 31.3% were aged 35–40 years; 36.7% of cohort members reported residing in rural areas, and 41.8% resided in urban areas in both 2005 and 2013. BMI at baseline indicated that 55.4% of cohort members were normal weight, 11.6% were underweight, 16.7% were overweight, and 16.2% were obese.

**Table 1:** Thai cohort member attributes at 2005 baseline and 8-year weight change between 2005 and 2013.

Cohort attributes at 2005 baseline (column %)	Eight-year weight change (row percent)				
	Δ ≥ −5%	−5% < Δ ≤ 5%	5% < Δ ≤ 10%	10% < Δ ≤ 20%	Δ > 20%
Overall (n = 27,003)	6.0	38.5	23.0	22.8	9.4
Male (45.0%, n = 12,157)	6.9	42.5	22.8	20.4	7.3
Female (54.9%, n = 14,846)	5.3	35.2	23.2	24.9	11.2
*Age groups*					
25–29 years (38.0%)	5.1	32.3	21.7	27.2	13.5
30–34 years (30.7%)	7.3	46.3	23.7	17.4	5.1
35–40 years (31.3%)	5.8	38.5	23.9	22.9	8.7
*Residence: 2005 and 2013*					
Rural–rural (36.7%)	6.2	39.2	22.4	22.8	9.2
Rural–urban (12.7%)	6.3	37.9	23.5	22.7	9.4
Urban–rural (7.4%)	5.8	36.8	23.6	22.6	10.9
Urban–urban (41.8%)	5.8	38.6	23.3	22.9	9.3
*Personal monthly income*					
<10,000 Baht (20.1%)	7.3	38.0	20.8	22.0	11.7
10,001–30,000 Baht (64.1%)	5.5	36.0	23.4	24.6	10.3
>30,000 Baht (15.7%)	5.8	41.2	23.7	21.7	7.5
*Body mass index category*					
Underweight (11.6%)	1.9	35.4	23.4	27.2	11.8
Normal (55.4%)	3.8	36.9	24.4	25.2	9.4
Overweight (16.7%)	7.7	44.6	21.5	19.6	6.3
Obese (16.2%)	15.0	42.4	20.8	16.4	5.1

Between 2005 and 2013, 6.0% of participants lost more than 5% of their baseline weight, 38.5% were stable, 23.0% had slight weight gain, 22.8% had moderate weight gain, and 9.4% had heavy weight gain (Table 1). Across the 8 years, weight maintenance (within 5% of baseline weight) was more common among males than females (42.5% vs. 35.2%) and was most common among participants aged 30–34 years (46.3%) and those in the highest income bracket (41.2%). There was little difference according to residence.

At the 8-year follow-up in 2013, 5.7% of cohort members reported ‘poor or very poor’ overall health, 8.9% reported ‘little or none’ for energy level, and 9.6% reported ‘quite a lot or extremely’ for emotional problems (Table 2).

**Table 2:** Longitudinal weight change and health outcomes by baseline body mass index categories for Thai cohort study participants between 2005 and 2013.

Weight change by 2013 outcomes^a^	Odds Ratio^b^ [95% CI] of adverse outcomes for 8-year longitudinal weight change by baseline body mass index categories^c^			
	Underweight (n = 2876)	Normal (n = 14,059)	Overweight (n = 4277)	Obese (n = 4079)
*Overall health* (*poor, 5.7%*)				
Δ ≤ −5%	1.22 [0.43–3.51]	1.33 [0.89–2.01]	1.36 [0.78–2.40]	1.33 [0.88–2.02]
−5% < Δ ≤ 5%	Reference	Reference	Reference	Reference
5% < Δ ≤ 10%	0.78 [0.56–1.08]	1.12 [0.96–1.31]	0.97 [0.73–1.29]	1.13 [0.89–1.45]
10% < Δ ≤ 20%	1.03 [0.69–1.55]	**1.39** [1.13–1.71]	**1.53** [1.01–2.29]	**2.47** [1.74–3.51]
Δ > 20%	1.08 [0.63–1.85]	**1.44** [1.09–1.90]	**1.82** [1.04–3.19]	**3.20** [2.00–5.16]
*Energy* (*little or none, 8.9%*)				
Δ ≤ −5%	0.84 [0.34–2.06]	**1.40** [1.06–1.85]	0.74 [0.46–1.18]	0.83 [0.61–1.12]
−5% < Δ ≤ 5%	Reference	Reference	Reference	Reference
5% < Δ ≤ 10%	0.78 [0.56–1.08]	1.12 [0.96–1.31]	0.97 [0.73–1.29]	1.13 [0.89–1.45]
10% < Δ ≤ 20%	1.12 [0.85–1.47]	**1.32** [1.14–1.52]	1.11 [0.84–1.47]	1.24 [0.97–1.61]
Δ > 20%	1.22 [0.85–1.74]	**1.36** [1.11–1.65]	1.30 [0.87–1.93]	**1.93** [1.38–2.71]
*Emotion* (*quite a lot, 9.6%*)				
Δ ≤ −5%	**1.98** [1.01–1.62]	**1.37** [1.05–1.80]	1.08 [0.72–1.62]	**1.49** [1.12–1.97]
−5% < Δ ≤ 5%	Reference	Reference	Reference	Reference
5% < Δ ≤ 10%	0.98 [0.72–1.35]	0.95 [0.82–1.10]	0.76 [0.56–1.04]	1.10 [0.84–1.45]
10% < Δ ≤ 20%	1.09 [0.82–1.47]	1.02 [0.89–1.19]	1.11 [0.84–1.48]	1.29 [0.97–1.71]
Δ > 20%	**1.64** [1.15–2.32]	1.07 [0.87–1.30]	1.26 [0.72–1.62]	**1.62** [1.07–2.46]

Bold values indicate statistically significance results when 95% confidence level does not contain the null hypothesis value.

CI, confidence interval.

a Adverse outcomes in 2013 were: ‘poor or very poor’ overall health; ‘little or none’ energy; and ‘quite a lot or extreme’ emotional problems.

b Adjusted for 2005 baseline age, sex, monthly personal income; and 2005–2016 residence.

c Each multivariate analysis excluded cohort members with adverse SF-8 health outcomes at 2005 baseline.

The findings from the logistic regression analyses are shown in Table 2. Moderate and heavy weight gain were both associated with an increased risk of reporting ‘poor or very poor’ overall health in 2013 among participants who had a normal BMI (AOR 1.39; 95% CI, 1.13–1.71] and AOR 1.44; 95% CI, 1.09–1.90, respectively), were overweight (AOR 1.53; 95% CI, 1.01–2.29 and AOR 1.82; 95% CI, 1.04–3.19, respectively), or had obesity (AOR 2.47; 95% CI, 1.74–3.51 and AOR 3.20; 95% CI, 2.00–5.16, respectively) in 2005. Eight-year weight change was also associated with an increased risk of reporting “little or no energy” in 2013. Among participants who had a normal BMI in 2005, weight loss >5% and weight gain >10% were both associated with an increased risk of reporting ‘little or no energy’ in 2013. Similarly, among participants who had obesity in 2005, >20% weight gain was also associated with having reduced energy levels (AOR 1.93; 95% CI, 1.38–2.71).

Cohort members who lost >5% of their initial weight at baseline had an increased risk of reporting ‘quite a lot’ or ‘extreme’ emotional problems (AOR 1.98; 95% CI, 1.01–1.62 among cohort members who were underweight in 2005; AOR 1.37; 95% CI, 1.05–1.80 among those who had a normal BMI in 2005, and AOR 1.49; 95% CI, 1.12–1.97 among participants who had obesity in 2005). On the other hand, cohort members who gained >20% of their baseline weight were more likely to report ‘quite a lot’ or ‘extreme’ emotional problems, but this association was statistically significant only among cohort members who were underweight in 2005 (AOR 1.64; 95% CI, 1.15–2.32) and those who had obesity in 2005 (AOR 1.62; 95% CI, 1.07–2.46).

## Discussion

Based on our longitudinal study, we were able to confirm that 8-year weight change associates with adverse health outcomes among adults in Thailand. The results also show that weight loss is associated with poor emotional health, especially among cohort members who were underweight at baseline. This study also found that weight gain was associated with poor overall health, poor emotional well-being, and lower levels of energy. These associations were particularly evident among Thai cohort participants who were overweight or had obesity at baseline.

Limited longitudinal evidence is available from low- and middle-income countries, but our findings were in line with those on the impact of weight change on adverse health from a 7-year cohort study of adults in Germany,^16^ an 8-year prospective cohort study among adults in Sweden,^17^ and a 10-year longitudinal study in the Netherlands^18^; these studies all revealed that weight gain was associated with lower functional health and quality of life, especially among adults who were overweight or had obesity.

Excess weight gain in early adulthood has been reported to have adverse effects in high-income Asian countries. A 5-year longitudinal cohort showed an effect of weight change and incident diabetes among Korean adults,^19^ and heavy weight gain has been associated with coronary heart diseases^20^ and cancers,^21^ even among non-obese Japanese adults. Our findings have added to the evidence that weight change has negative effects on health outcomes in a middle-income Asian setting.

Among the Thai Cohort Study cohort, young females gained the most weight between 2005 and 2013, so they are at an increased risk of poorer health outcomes, a finding that is consistent with the findings from the national health examination survey on the high prevalence of overweight and obesity among females, who already have an increased metabolic risk among the Thai population.^22^ This group could be targeted with gender-specific campaigns to promote gradual, well-managed (rather than rapid) weight loss. The population, more generally, could be alerted to the deleterious effects of rapid weight change on physical and emotional health and energy levels, as a better understanding of these health risks could improve weight management strategies. This study points to the importance of preventing weight gain and future non-communicable diseases by focusing on weight maintenance and early healthy lifestyles throughout the lifecourse.^23^

In interpreting our findings, some limitations should be considered. Notably, weight and height in this study were self-reported. However, another related study based on the same study population noted that correlations between measured and self-reported weight and height were high in both sexes, ranging from 0.91 to 0.95.^24^ For longitudinal observation, weight change was observed from BMI reported at the 2005 baseline and 2013 follow-up, so there could be fluctuation in weight between these time points that were not accounted for in the analyses. We also could not assess if the weight change was intentional or not. In the final analyses, we excluded cohort members who reported adverse outcomes at baseline to minimize the reverse causation between weight change and health effects.

Our study found that weight change, in particular weight gain, was associated with negative health outcomes, and this effect appeared to increase at higher levels of body size. Future health promotion initiatives to improve health outcomes should focus on preventing weight gain and subsequent adverse health effects, especially among young adults, who appear to have larger fluctuations in weight than older adults.

## Conflicts of interest

None declared.
